# *Mycobacterium tuberculosis* Infection of Retinal Endothelial Cells Induces Interferon Signaling Activation: Insights Into Tubercular Retinal Vasculitis

**DOI:** 10.1167/iovs.66.9.48

**Published:** 2025-07-16

**Authors:** Ikhwanuliman Putera, Sigrid M. A. Swagemakers, Nicole M. A. Nagtzaam, Conny van Holten-Neelen, Rina La Distia Nora, Jurriaan E. M. de Steenwinkel, Saskia M. Rombach, P. Martin van Hagen, Willem A. Dik

**Affiliations:** 1 Department of Ophthalmology, Faculty of Medicine, University of Indonesia–Cipto Mangunkusumo Hospital, Jakarta, Indonesia; 2 Department of Ophthalmology, Erasmus University Medical Center, Rotterdam, the Netherlands; 3 Department of Internal Medicine Section Allergy and Clinical Immunology, Erasmus University Medical Center, Rotterdam, the Netherlands; 4 Medical Immunology Laboratory, Department of Immunology, Erasmus University Medical Center, Rotterdam, the Netherlands; 5 Department of Pathology and Clinical Bioinformatics, Erasmus MC University Medical Center Rotterdam, Rotterdam, the Netherlands; 6 Department of Medical Microbiology and Infectious Diseases, Erasmus University Medical Center, Rotterdam, the Netherlands; 7 Department of Immunology, Faculty of Medicine, Chulalongkorn University, Bangkok, Thailand; 8 Department of Internal Medicine, Faculty of Medicine, University of Indonesia–Cipto Mangunkusumo Hospital, Jakarta, Indonesia; 9 Reinier Haga Medisch Diagnostisch Centrum, Medical Immunology Laboratory, Delft, the Netherlands

**Keywords:** immune response, interferon signaling, ocular tuberculosis, retinal endothelial cells, retinal vasculitis

## Abstract

**Purpose:**

Ocular tuberculosis (OTB) has diverse clinical presentations, among which choroidal granuloma and tubercular retinal vasculitis (TRV) are recognized as typical phenotypes. The potential role of human retinal endothelial cells (RECs) in regulating inflammation in response to *Mycobacterium tuberculosis* (*Mtb*) infection, particularly relevant in cases of TRV, remains elusive. This study investigated the cellular defense of *Mtb*-exposed RECs.

**Methods:**

Human RECs, as a TRV model, were exposed to either live H37Rv *Mtb* or heat-killed (HK)-H37Rv *Mtb*. Total RNA and culture supernatants were collected 24 hours post-exposure, and cellular responses were characterized using RNA sequencing and bioinformatics analysis.

**Results:**

Both live and HK-*Mtb* were internalized by RECs, but only live *Mtb* induced a robust transcriptional response, with 322 differentially expressed genes. Live *Mtb* infection, but not HK-*Mtb* exposure, induced a dominant interferon (IFN) signaling response, particularly type I IFN activation. This was validated by real-time polymerase chain reaction (RT-PCR) for 10 IFN-inducible genes. Network analysis suggested a role of RECs in leucocyte recruitment and activation. Although HK-*Mtb* increased the production of CCL2, IL-6, and IL-8, this was not observed with live *Mtb* infection. Instead, live *Mtb* infection led to elevated CXCL10 and IP-10 production.

**Conclusions:**

RECs elicit a robust immune response to *Mtb* infection. IFN signaling activation was observed in live *Mtb*-infected RECs but not in HK-*Mtb*-exposed RECs. These findings provide insights into TRV pathogenesis and offer clues for potential biomarkers that may help differentiate TRV caused by active infection, which requires antitubercular treatment, from cases without active infection, which require immunosuppressive therapy alone.

Ocular tuberculosis (OTB) is a common cause of infectious uveitis, particularly in high tuberculosis-burden settings.^1^^,^^2^ The clinical presentation of OTB can be diverse, but choroidal granuloma and retinal vasculitis are recognized as typical phenotypes.^3^ *Mycobacterium tuberculosis* (*Mtb*) can disseminate from the lungs to the eyes through hematogenous spread.^4^^,^^5^ Histopathological studies have identified the presence of *Mtb* within the retinal pigment epithelial (RPE) layer in cases of OTB.^6^ To delineate the host–pathogen interaction, in vitro studies have utilized RPE–*Mtb* infection models to characterize the cellular response *Mtb* elicits in RPE cells.^7^^–^^9^ These studies strongly suggest that the RPE, as part of the outer blood–retinal barrier (oBRB),^10^ likely contributes to the development of choroidal granuloma formation and/or choroiditis in OTB. However, the immunopathobiology of retinal vasculitis, also referred to as tubercular retinal vasculitis (TRV),^11^ that can occur in OTB was not addressed directly by these experiments.^7^^–^^9^

Retinal endothelial cells (RECs) are the primary component of the inner blood–retinal barrier (iBRB)^10^ and a relevant cell type in the context of TRV pathogenesis.^12^ Our current understanding of endothelial cells supports their active role in immunity, as they exhibit a wide range of innate immune response capacities.^13^ Evidence of a direct role for *Mtb* in triggering TRV is supported by already old observations that injection of live or dead *Mtb* into the carotid artery of rabbits results in TRV.^14^ Positive *Mtb* polymerase chain reaction (PCR) from ocular fluid specimens from patients with TRV, as well as reports of intraretinal granuloma formation adjacent to areas of vasculitis, have been described.^15^^,^^16^ Therefore, studies that investigate the interaction between REC and *Mtb* are essential, as they could offer valuable immunobiological insights that may improve the diagnosis and treatment of TRV.

The present study aimed to characterize the cellular response of human RECs to *Mtb* and to examine whether this response differs between live and heat-killed (HK) *Mtb*. Understanding this may help identify reliable biomarkers to distinguish TRV cases requiring antitubercular treatment (ATT) in addition to immunosuppressive therapy due to viable *Mtb* infection from cases of retinal vasculitis potentially caused by dead bacteria, which may not necessitate ATT but rather immunosuppressive therapy alone.

## Methods

### Cell Culture

All experiments were performed with the human REC cell line ACBRI 181 (Cell Systems, Kirkland, WA, USA), at passages 11 to 13. The RECs were cultured in the EGM Endothelial Cell Growth Medium BulletKit (CC-3124; Lonza, Basel, Switzerland) and maintained in an incubator (37°C, 5% CO_2_). The culture medium was refreshed every other day until the cells were exposed to live or dead (heat-killed) *Mtb*. Prior to cell seeding, culture flasks were precoated with 0.2% gelatin for at least 30 minutes.

### Exposure of Human RECs to *Mtb*

RECs were resuspended in fresh culture medium (1.5 mL/well) without antibiotics 1 day prior to exposure with *Mtb* (ATCC 27294 strain, H37Rv). Human RECs (0.5 × 10^6^ cells/well) were seeded overnight on gelatin-precoated chamber slides (Nunc Lab-Tek Chamber Slide System, 177429PK; Thermo Fisher Scientific, Waltham, MA, USA). The culture medium was refreshed before *Mtb* exposure. A pre-prepared *Mtb* suspension stored at −80°C was thawed at room temperature for 30 minutes and centrifuged (14,000 rpm, 10 minutes), and the supernatant was discarded. The pellet was resuspended in phosphate-buffered saline (PBS). Cells were exposed to a multiplicity of infection of 10 bacteria per cell (10:1) for 3 hours. Following a 3-hour incubation period, the medium was discarded, and cells were gently washed twice with 1 mL PBS. The cells were then incubated in fresh human REC culture medium without antibiotics for 24 and 48 hours.

HK-*Mtb* (strain H37Rv) were prepared after thawing and centrifuging a pre-prepared *Mtb* suspension, as described above. The bacterial cells were resuspended in 1 mL of PBS and subsequently aliquoted into centrifuge tubes and heat-killed in a heat block set at 85°C for 45 minutes with gentle agitation, following a modified protocol from a previous study.^17^ Non-viability of the HK-*Mtb* was confirmed by culturing the suspension (undiluted) on Middlebrook 7H10 OADC (Oleic Albumin Dextrose Catalase) media agar plates (Thermo Fisher Scientific) for 12 weeks at 37°C, with no bacterial growth observed. RECs were exposed to HK-*Mtb* using the same quantity of enumerated bacteria as those used for infection with live *Mtb* and incubated for 24 hours in growth medium without antibiotics. Non–*Mtb*-exposed RECs, otherwise cultured in the same manner, served as controls. Experiments with *Mtb* (live and HK) were performed in a Biosafety Level 3 (BSL-3) laboratory dedicated to tuberculosis research at Erasmus MC. The data generated for this study were the result of at least three independent experiments.

### Microscopy and Colony-Forming Unit Enumeration

Human RECs seeded in chamber slides were prepared for acid-fast bacilli (AFB) Kinyoun staining under three conditions: (1) exposure to live *Mtb* (*t* = 24 hours), (2) exposure to HK-*Mtb* (*t* = 24 hours), and (3) non–*Mtb*-exposed REC controls. After washing and air-drying, the chamber slide walls were removed, and the cells were fixed on a heated block at 100°C for 10 minutes prior to AFB Kinyoun staining. Images from the slides were acquired using a ZEISS Axiocam 305 color microscopy camera (Carl Zeiss Microscopy, Jena, Germany). A colony-forming unit (CFU) assay was conducted by discarding the culture supernatants, washing twice with 1 mL PBS, and treating each well of the chamber slides with 0.5% Triton X-100 for 5 minutes. From the cell lysates, serial dilutions were prepared in PBS and plated on the Middlebrook 7H10 OADC agar plates. After 4 weeks of incubation, CFUs were manually counted.

### RNA Isolation and Transcriptome Profiling

Total RNA was isolated from the RECs seeded in chamber slides with the FastRNA Pro Blue Kit, according to the manufacturer's protocol (MP Biomedicals, Irvine, CA, USA). After washing, 0.5 mL RNApro solution was added to the chamber slide wells and gently mixed. The lysates from two different wells were then combined and transferred to a dedicated Lysing Matrix-B tube, resulting in a total of 1 mL in each tube. RNA yield was measured using a NanoDrop ND1000 spectrophotometer (NanoDrop Technologies, Wilmington, DE, USA). The quality and integrity of the RNA were determined with a 2100 BioAnalyzer (Agilent, Santa Clara, CA, USA), yielding an average RNA integrity number equivalent (RINe) score of 6. Consequently, we proceeded with the QuantSeq 3′ mRNA-Seq protocol (Lexogen, Vienna, Austria). Sequencing libraries were prepared at the Genomics Facility, Erasmus MC, Rotterdam, the Netherlands, and sequenced at 2 × 150 bp on a NextSeq 500 sequencer (Illumina, San Diego, CA, USA).

### Bioinformatics Analysis

RNA sequencing (RNA-seq) count data was normalized within the R package DESeq2 (R Foundation for Statistical Computing, Vienna, Austria).^18^ Principal component analysis (PCA) was performed to assess the effect of the different REC treatments: 24-hour exposure to (1) live *Mtb*, (2) HK-*Mtb*, and (3) non–*Mtb*-exposed controls. Differential gene expression results were analyzed using Ingenuity Pathway Analysis (IPA; QIAGEN, Hilden, Germany) by applying cutoff-adjusted *P* values of 0.01. Canonical pathways were sorted and filtered based on a Benjamini–Hochberg *P* value threshold of 0.05 and a *z*-score cutoff of 3.0. We also evaluated the protein network, master regulators, and regulator effects using IPA. The regulator effects analysis module enables the identification of predicted activated and inhibited upstream regulators, along with their associated predictions of phenotypic or functional outcomes.

To evaluate the relevance of the cellular responses of human RECs compared to other cell types involved in the immunopathobiology of OTB and pulmonary TB, we retrieved and analyzed RNA-seq datasets from other *Mtb* infection experiments. These included in vitro studies using human RPE cells, lung endothelial cells, lung (parenchyma) epithelial cells, and lung alveolar macrophages. RNA-seq data from *Mtb*-infected RPE cells were retrieved from our previous work (primary RPE cells [OZR1], DESEQ2, adjusted *P* < 0.01^7^). For the other cell types, RNA-seq datasets were retrieved from a study by Maertzdorf et al.^19^ (GSE112483), which performed *Mtb* infection experiments using lung endothelial cells, lung epithelial cells, and lung alveolar macrophages derived from human lung tissue biopsies. All additional RNA-seq datasets that we retrieved (*Mtb*-infected OZR1 cells, *Mtb*-infected lung epithelial cells, *Mtb*-infected lung endothelial cells, and *Mtb*-infected lung alveolar macrophages) were generated from 24 hours of *Mtb* infection experiments using strain H37Rv. Canonical pathways identified in the human REC dataset were compared with those from RPE cells, lung endothelial cells, lung epithelial cells, and lung alveolar macrophages using a comparison analysis module in IPA. We analyzed pathways with activation *z*-scores > 3 or < −3.

### Real-Time Quantitative Reverse Transcriptase Polymerase Chain Reaction for Selected Interferon-Inducible Genes

Total RNA (0.45 µg) was first reverse-transcribed into complementary DNA (cDNA) using random primers (Thermo Fisher Scientific). We measured the expression levels of 10 IFN-inducible genes (ISGs)—*FCGR1B*, *GBP1*, *IFIT2*, *IRF7*, *MyD88*, *Mx1*, *SERPING1*, *STAT1*, *TLR8*, and *UBE2L6*—that were previously found to be upregulated in RPE cells upon *Mtb* infection and have shown clinical relevance in OTB diagnosis.^7^^,^^20^^,^^21^ The commercially available primer–probe combinations used to detect the ISGs and the housekeeping gene *ABL* are listed in Supplementary Table S1. Thermocycling was performed on the QuantStudio 5 Flex Real-Time PCR machine (Applied Biosystems, Waltham, MA, USA) using TaqMan Universal PCR Master Mix (4,366,072; Applied Biosystems). The calculation of relative gene expression levels was performed by normalizing to the expression of the housekeeping gene *ABL*, using the 2^–^^ΔΔCt^ method.

### Cytokine Analysis

Culture supernatants collected from chamber sides (*t* = 24 hours) were analyzed using a customized 12-plex human Luminex discovery immunoassay (R&D Systems, Minneapolis, MN, USA) for CCL2, CCL5, CXCL9, CXCL10/IP-10, IFN-α, IFN-γ, IL-6, IL-8, IL-10, IL-12, TNF-α, and VEGF-A, according to the manufacturer's instructions. The selection of these proteins was based on a previous study with *Mtb*-infected RPE cells (OZR1) that found upregulation of these proteins upon infection.^7^ Analysis of the supernatants was conducted after passing them through an Eppendorf membrane filter at a BSL-3 laboratory. Assay measurement was conducted on a MAGPIX system (Luminex, Austin, TX, USA), and data were analyzed using Bio-Plex Manager MP software (Bio-Rad, Hercules, CA, USA).

### Statistical Analysis

All graphs and statistical analyses were performed using Prism 9.0.0 for Windows (GraphPad, Boston, MA, USA). Comparison analyses were conducted using the Kruskal–Wallis test, followed by post hoc uncorrected Dunn's test. *P* < 0.05 was considered statistically significant.

## Results

### Live and Heat-Killed *Mtb* Are Internalized by Human RECs

**Figure 1.**
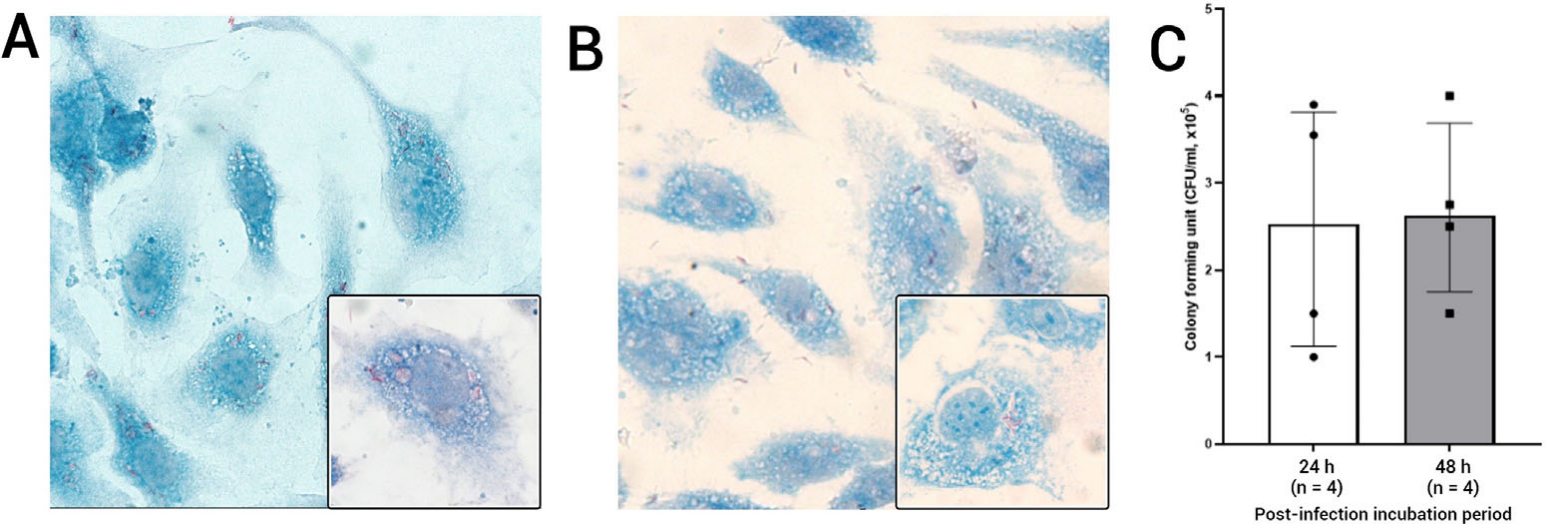
Internalization of *Mtb* in human RECs. (**A**) Microscopy image of human RECs 24 hours after infection with live H37Rv *Mtb* (AFB Kinyoun staining: 40× magnification, with the inset image at 100× magnification). (**B**) Microscopy image of human RECs 24 hours after exposure to heat-killed H37Rv *Mtb* (AFB Kinyoun staining: 40× magnification, with the inset image at 100× magnification). (**C**) CFU assays were performed at 24 hours and 48 hours after infection with live *Mtb* (*n* = 4 experiments for each *bar*). The *box* indicates the median CFUs, and the *bars* indicate the interquartile range.

Microscopic examination of AFB Kinyoun-stained slides revealed that human RECs exposed to live *Mtb* internalized the bacteria (Fig. 1A). The same was observed when RECs were exposed to HK-*Mtb* (Fig. 1B). At 24 and 48 hours after exposure of RECs to live-*Mtb*, we observed median CFU counts per cell (×10^5^) of 2.5 and 2.6, respectively (Fig. 1C). This indicates that *Mtb* infects REC and can survive for at least 48 hours in human RECs.

### Host Response of Human RECs Exposed to Live *Mtb* Compared to Those Exposed to HK-*Mtb*

**Figure 2.**
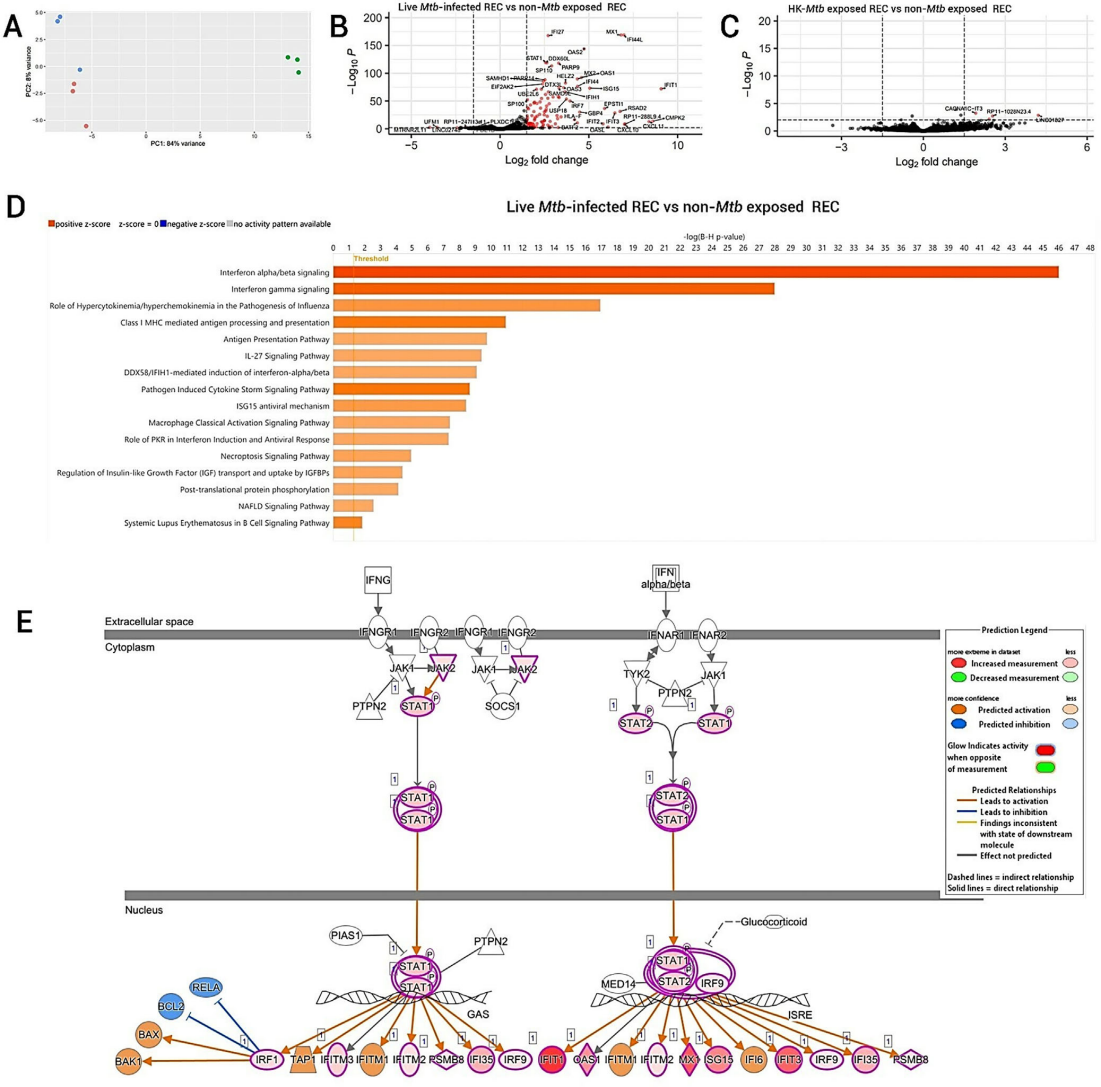
Human REC responses characterization after infection with live *Mtb* (strain H37Rv) or exposure to heat-killed *Mtb* (strain H37Rv) for 24 hours based on RNA-seq data and bioinformatics analysis. (**A**) PCA of the overall gene expression profiles in RECs after exposure to live *Mtb* (*green dots*, *n* = 3 experiments), exposure to HK-*Mtb* (*blue dots*, *n* = 3 experiments), or non–*Mtb*-exposed controls (*red dots*, *n* = 3 experiments). (**B**) Volcano plot of differentially expressed genes of human RECs upon live *Mtb* infection. (**C**) Volcano plot of differentially expressed genes of human RECs following exposure to heat-killed *Mtb*. (**D**) Canonical pathways regulated in human RECs upon live *Mtb* infection (sorted by Benjamini–Hochberg *P*-value cutoff of 0.05 and *z*-score cutoff of 3.0 in IPA). *Orange* indicates positive *z*-scores (activated pathways), *white* indicates a *z*-score of 0 (no activation or inhibition), *blue* indicates negative *z*-scores (inhibited pathways), and *gray* indicates pathways for which no activity pattern could be determined. Note that all pathways depicted in this figure are *orange*, indicating positive *z*-scores (activation). (**E**) Visualization of interferon signaling pathway activated in human RECs upon live *Mtb* infection. The different colored gene nodes mean the following: *Red nodes* indicate that the RNA showed increased expression, *orange nodes* indicate that these molecules are predicted to be activated, and *blue nodes* indicate that these molecules are predicted to be inhibited. The different line coloring means the following: *Orange lines* indicate a predicted activating effect, *blue lines* indicates a predicted inhibitory effect, and *gray lines* indicate an unpredictable effect. The pathways shown in (**D**) and (**E**) were generated through the use of QIAGEN IPA.

Next, we characterized the host response of human RECs following exposure to live and HK-*Mtb*. A PCA revealed profound differences in the overall gene expression patterns between the different exposure conditions. The difference in gene expression pattern was most pronounced for RECs that were exposed to live *Mtb*, whereas the RECs exposed to HK-*Mtb* clustered more closely to non-*Mtb* exposed controls (Fig. 2A). Compared to non-exposed RECs, a total of 322 differentially expressed genes (DEGs) were identified in RECs exposed to live *Mtb* and nine DEGs in RECs exposed to HK-*Mtb* (Figs. 2B, 2C). These findings reflect a stronger regulation of gene expression in human RECs following exposure to, and subsequent infection with, live *Mtb*, with only minimal changes occurring upon exposure to HK-*Mtb*.

Genes that were differentially expressed under both exposure conditions were put into IPA to identify the signaling pathways involved in the host response to *Mtb* infection. In addition to identifying signaling pathways, the bioinformatics analyses using IPA also provided global insights into the cellular responses of RECs to *Mtb* infection by identification of activated or inhibited upstream regulators, as well as associated diseases and functions. Furthermore, network analysis and protein–protein interaction maps can be generated and visualized based on observed DEGs. The top canonical pathway activated in human RECs following live *Mtb* infection was type-1 IFNα/β signaling, followed by IFN-γ signaling (Figs. 2D, 2E). No canonical pathways could be identified from the DEGs found in HK-*Mtb*–exposed RECs.

**Figure 3.**
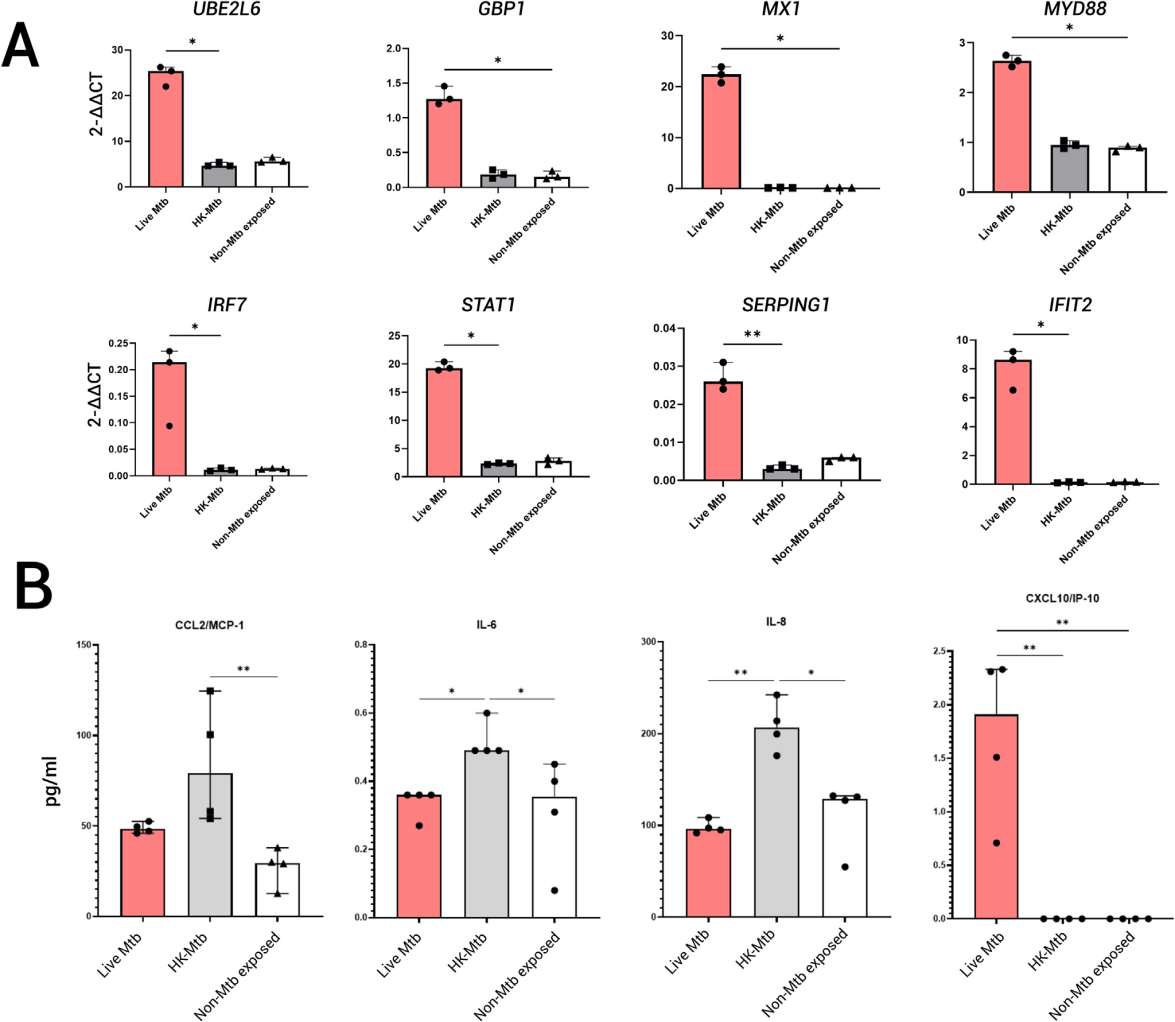
Measurement of selected IFN-inducible gene expressions and proteins contained in cell culture supernatants. (**A**) Bar graphs represent the expression levels of IFN-inducible genes as measured with RT-PCR (*n* = 3 per experimental group, performed in duplo). (**B**) Bar graphs represent the level of four cytokines and chemokines measured in the culture supernatants (*n* = 4 per experimental group). The height of the bars in (F) and (G) represent the median value, and the *upper* and *lower horizontal bars* represent 95% confidence intervals. *P <0.05, **P < 0.01.

To independently validate the activation of IFN signaling in *Mtb*-infected RECs, as observed in the bioinformatics analysis, we conducted RT-PCR for 10 IFN-inducible genes. Two genes (*FCGR1B* and *TLR8*) were undetectable, but expression of the remaining eight genes (*GBP1*, *IFIT2*, *IRF7*, *MyD88*, *Mx1*, *SERPING1*, *STAT1*, and *UBE2L6*) was highly upregulated only in live *Mtb*-infected cells (Fig. 3A).

Next, we measured 12 cytokines/chemokines in culture supernatants. Of these, CCL2, IL-6, IL-8, and CXCL10/IP-10 were detectable. Interestingly, in culture supernatants from RECs exposed to HK-*Mtb*, significantly higher levels of CCL2, IL-6, and IL-8 were found compared to culture supernatants from non-exposed RECs, whereas this difference did not exist between the culture supernatants from RECs exposed to live *Mtb* and the non-exposed RECs. Likewise, IL-6 and IL-8 levels were significantly higher in culture supernatants from RECs exposed to HK-*Mtb* than from RECs exposed to live *Mtb*. In contrast, the IFN-inducible chemokine CXCL10/IP-10 was only elevated in culture supernatant from RECs exposed to live *Mtb* (Fig. 3B), which corroborates the strong activation of IFN signaling in human RECs upon *Mtb* infection (Figs. 2D, 2E, 3A). Of note, CCL2, IL-6, and IL-8 were not found among the DEGs in human RECs exposed to HK-*Mtb* at 24 hours post-infection.

**Figure 4.**
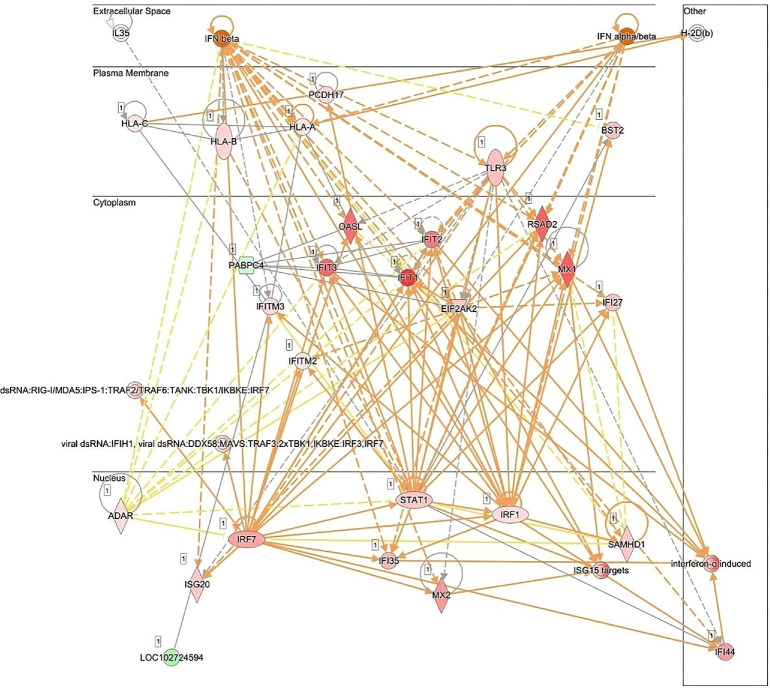
Network analysis of the host response in human RECs upon *Mtb* infection. Ingenuity-based pathway analysis of genes that were differentially expressed between live *Mtb*-infected RECs and REC controls (*t* = 24 hours). Depicted is the predicted network visualized in a subcellular arrangement. *Symbols* depict the genes, and the coloring indicates the following: *Red genes* indicate that the RNA was measured at an increased expression level, *green genes* indicate that the RNA was measured at a decreased level, and *orange* indicates genes that are predicted to be activated. The different line *coloring* means the following: *Orange lines* indicate a predicted activating effect, *yellows lines* indicate an inconsistency with what was actually measured for the respective gene, and *gray lines* indicate an unpredictable effect. *Solid lines* are indicative of a direct effect, and the *dashed lines* indicate an indirect effect. The network was generated through the use of QIAGEN IPA.

**Figure 5.**
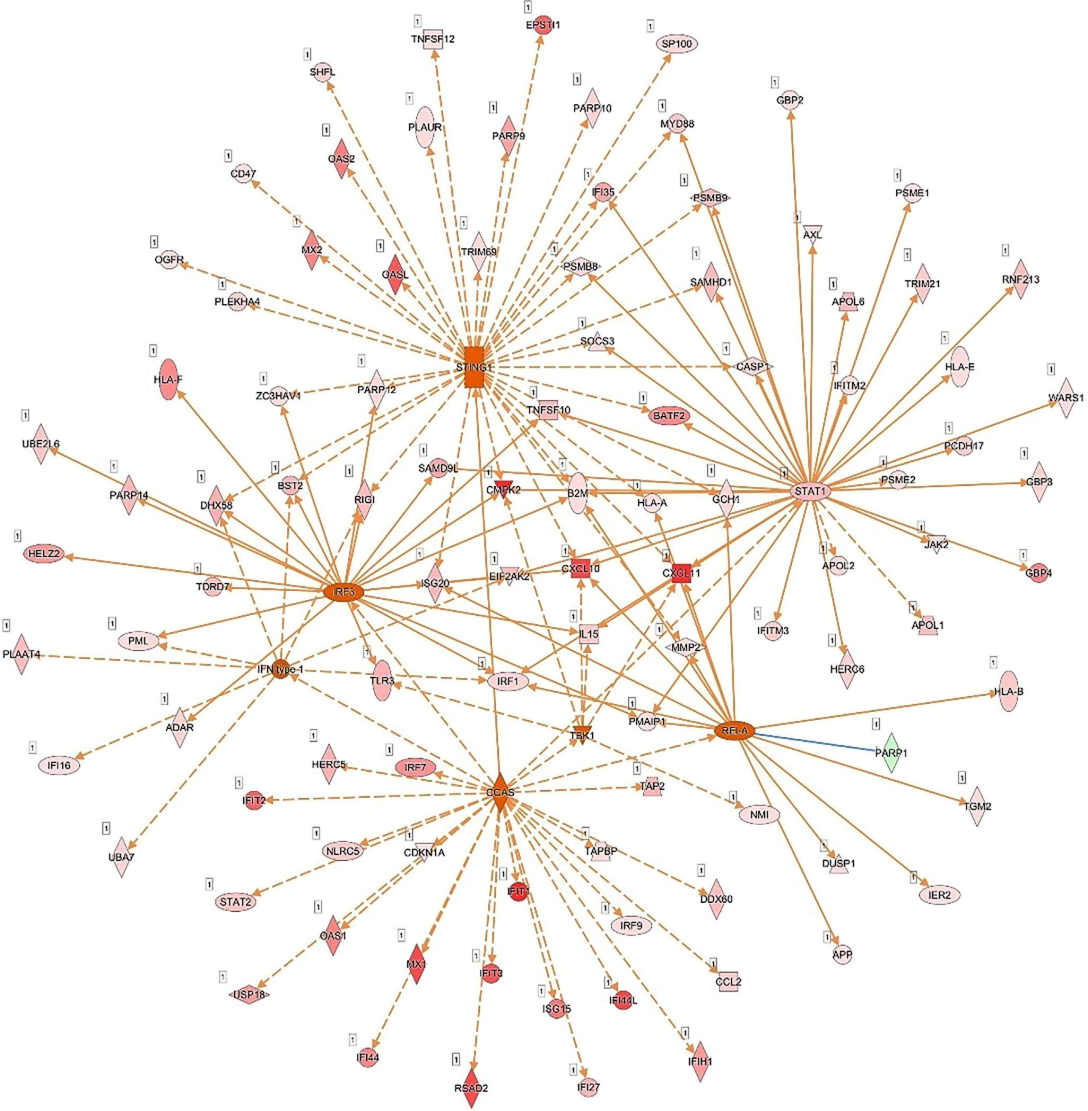
Network visualization of cGAS, identified as being among the top master regulators based on the DEGs in human RECs following live *Mtb* infection. *Symbols* depict the molecules and the *coloring* indicates the following: *Red molecules* were measured at an increased level, *green molecules* were measured at a decreased level, and *orange molecules* were predicted to be activated. The *orange line* indicates the activating effect. *Solid lines* indicate a direct effect, and *dashed lines* indicate an indirect effect. The network was generated through the use of QIAGEN IPA.

Network analysis corroborated the role of IFN signaling-associated molecules in the response of human RECs to live *Mtb* infection (Fig. 4). The presented network includes 27 focus molecules and highlights antimicrobial response, immunological disease, and inflammatory response as the top predicted diseases and functions. Notably, IPA identified cyclic GMP–AMP synthase (cGAS) as one of the top three master regulators in the dataset (Fig. 5). Among the visualized network, four key genes (*cGAS*, *STING1*, *IRF3*, and *STAT1*) were identified as dominant nodes. Several ISGs, including *STAT1*, *UBE2L6*, *MX1*, *IRF7*, and *IFIT2*, were validated by RT-PCR, whereas CXCL10 protein was measured in the culture supernatant. Additionally, the top 10 predicted regulatory effect networks are listed in the Table, and they indicate that *Mtb*-infected RECs play a role in the recruitment and activation of leucocytes. Although IFN-β was not among the top 10 predicted regulatory effects, it was predicted to be activated and implicated in the recruitment of leukocytes (Supplementary Figure S1). Supplementary Table S2 lists the predicted master regulators associated with the expressed genes.

**Table.** Top 10 Ingenuity-Based Regulator Effect Predictions Derived From DEGs in Human RECs Following Live *Mtb* Infection

No.	Consistency Score	Node Total, *n*	Regulators	Target Molecules in Dataset	Diseases and Functions
1	3.638	19	poly rI:rC-RNA	APP, CCL2, CSF1, CXCL10, CXCL11, EGR1, IL15, IRF7, LGALS9, MDK, MMP2, MYD88, PTGS2, RIGI, SERPINE1, STAT1, TLR3	Recruitment of leukocytes
2	3.606	15	TNF	CCL2, CCN1, CD47, CD55, CSF1, CXCL10, CXCL11, IL15, MYD88, PTGS2, RIGI, STAT1, TNFSF12	Recruitment of mononuclear leukocytes
3	3.464	14	ETV3	BST2, EIF2AK2, IFI16, IFIH1, IFIT1, IFIT5, MX1, OAS1, OAS3, OASL, PLSCR1, RSAD2	Replication of viral replicon
4	3.464	14	ETV3	BST2, EIF2AK2, IFI16, IFIH1, IFIT1, IFIT5, MX1, OAS1, OAS3, OASL, PLSCR1, RSAD2	Viral life cycle
5	3.464	14	IFNA2	BST2, CNP, EIF2AK2, HERC5, HERC6, IFIH1, IFIT1, MX1, PML, RSAD2, TLR3, TRIM22	Production of virus
6	3.464	14	lipopolysaccharide	CCL2, CCN1, CD47, CD55, CSF1, CXCL10, CXCL11, IL15, MYD88, PTGS2, RIGI, STAT1	Recruitment of mononuclear leukocytes
7	3.333	11	USP18	CASP1, CCL2, CXCL10, IFIH1, IL15, IRF1, RIGI, STAT1, TNFSF10	Activation of leukocytes
8	3.317	13	IFNG	CCL2, CD55, CSF1, CXCL10, CXCL11, IL15, MYD88, PTGS2, RIGI, STAT1, TNFSF12	Recruitment of mononuclear leukocytes
9	3.182	10	RIGI	CCL2, CXCL10, CXCL11, IRF7, PTGS2, STAT1, TLR3, TNFSF12	Recruitment of leukocytes
10	3.182	10	USP18	CASP1, CCL2, CXCL10, IFIH1, IL15, RIGI, STAT1, TNFSF10	Activation of antigen-presenting cells

### Comparative Prediction of Cellular Responses Among Human RECs, Human RPEs, and Other Lung-Derived Cells Upon *Mtb* Infection

In this analysis, we aimed to compare (1) the cellular response of *Mtb*-infected human RECs with *Mtb*-infected human RPE cells, and (2) the cellular response of *Mtb*-infected human RECs with *Mtb*-infected human pulmonary cells. Specifically, we sought to compare human RECs to other endothelial cell (e.g., lung endothelial cells) to explore whether the observed response is unique to human RECs or shared with other organ-specific endothelial cells. Additionally, we compared the immune responses across these cell types (non-hematological cells) that are mostly involved in OTB and pulmonary TB immunopathobiology.

**Figure 6.**
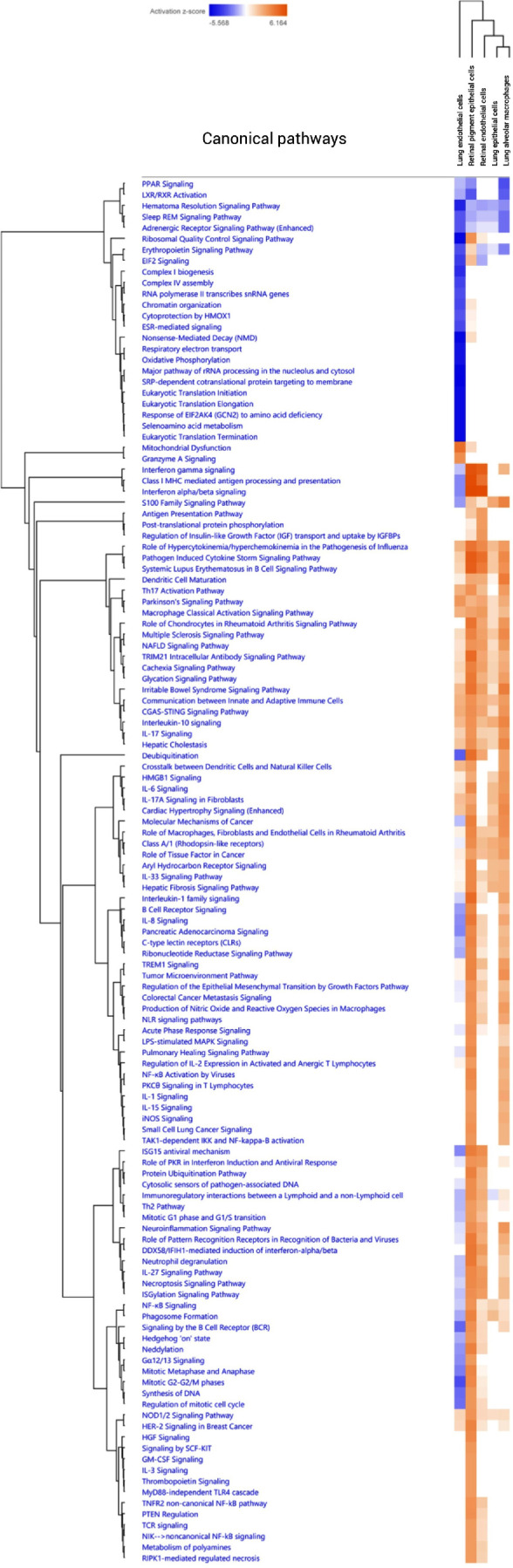
Comparison of canonical pathways inhibited or activated in human RECs, RPE cells, lung endothelial cells, lung epithelial cells, and lung alveolar macrophages following *Mtb* infection using unsupervised hierarchical clustering sorting method in IPA. The RPE cell dataset was obtained from a study by La Distia Nora et al.^7^ Lung endothelial cell, lung epithelial cell, and lung alveolar macrophage datasets were retrieved from a study by Maertzdorf et al.^19^ (GSE112483). The heat map was generated through the use of QIAGEN IPA.

Based on the hierarchical sorting of Ingenuity Canonical Pathways, the cellular response of human RECs upon *Mtb* infection closely resembled that of human RPE cells (Fig. 6). Furthermore, both human RECs and human RPE cells exhibited similarities to lung alveolar macrophages when infected with *Mtb*, although a stronger IFN response occurred in both RECs and RPE in comparison to the pulmonary arterial cells (Fig. 6). Activation of several pathways was observed in all cell types, including the pathogen-induced cytokine storm signaling pathway, macrophage classical activation signaling pathway, IL-10 signaling pathway, communication between innate and adaptive immune cells, and the cGAS–STING pathway. Although lung endothelial cells displayed a response to *Mtb* infection with commonalities to the other cell types, clear differences were also apparent in comparison to the responses observed in human RECs and the other cell types. For example, IPA predicts that pathways critical for TB defense mechanisms, such as phagosome formation, IL-8 signaling, interleukin-1 family signaling, and the IL-27 signaling pathway, are inhibited in lung endothelial cells but not in other cell types.

## Discussion

To our knowledge, this study is the first to demonstrate that *Mtb* can infect human RECs in an in vitro infection model. Bioinformatics analysis of the transcriptomics dataset provided global insights into the cellular response of human RECs to *Mtb* infection. This infection is associated with strong activation of IFN signaling, as became evident from gene transcriptional alterations and extensive CXCL10/IP-10 secretion. In contrast, although HK-*Mtb* was internalized by human RECs, possibly through phagocytosis, no specific signaling pathways could be identified on the basis of alterations in the gene transcriptional program. However, we did observe a marked increase in the production of three pro-inflammatory cytokines/chemokines (CCL2, IL-6, and IL-8).

Multiple observations suggest that type 1 IFN expression may impair the host's ability to combat *Mtb*,^22^^–^^24^ although others have highlighted the importance of type 1 IFN responses in controlling the proliferation, differentiation, activation, and maturation of various leukocyte populations, including the stimulation of IFN-γ production by natural killer (NK) cells and T lymphocytes that is required to combat *Mtb*.^25^ The observed high expression of the chemokine CXCL10/IP-10, which occurred selectively in response to live *Mtb* infection, suggests a central role of human RECs in TRV pathogenesis. Previous experiments have demonstrated that *Mtb*-infected dendritic cells produce CXCL10, which acts as a chemotactic factor for NK cells and T lymphocytes (both CD4^+^ and CD8^+^), that contribute to the formation of TB granulomas at the infection site.^25^ In support of this, our bioinformatic analysis predicted that human RECs may contribute to the recruitment and activation of leukocytes at the infection site (Table). Interestingly, our present observations with HK-*Mtb* indicate that *Mtb* antigens can induce an inflammatory response to a lesser extent. This is evidenced by the production of CCL2, IL-6, and IL-8, even though only few significant alterations in gene expression were observed (not including the genes encoding CCL-2, IL-6, and IL-8), and no pathways were predicted to be activated or inhibited. This may, however, be related to the time point (24 hours after exposure) chosen for the RNA-seq analysis in this study. Therefore, we cannot exclude that HK-*Mtb* did induce significant transcriptional alterations in the RECs earlier after exposure. Nevertheless, our data do support that *Mtb* antigens, even in the absence of live *Mtb*, may be clinically sufficient to trigger retinal vasculitis. However, only infection with live *Mtb* activated IFN signaling in RECs.

One potential mechanism behind the selective activation of IFN signaling in response to live *Mtb* infection, as opposed to HK-*Mtb* exposure, could involve the cGAS/STING pathway.^26^^,^^27^ This pathway necessitates the binding of *Mtb* DNA to cGAS in the cytosol of the infected cells,^26^^–^^28^ which likely occurs when replicating *Mtb* is recognized intracellularly. The type 1 IFN response, especially IFN-β, is dose-dependently activated by *Mycobacterial* extracellular DNA (eDNA).^29^ In case of dead, rather than live, *Mycobacteria*, lower amounts of eDNA will be sensed by cGAS, potentially limiting activation of the cGAS/STING pathway and, consequently, the type 1 IFN response.^29^ Notably, in our experiment with live *Mtb*-infected RECs, activation of the cGAS/STING pathway was also predicted through IPA analysis (canonical pathway *z*-score = 2.6, *q*-value = 0.0004) (Fig. 6, Supplementary Fig. S2). The activation of the cGAS/STING signaling pathway is predicted across the different cell types upon *Mtb* infection (Fig. 6), although the specific genes involved may vary between cell types (Supplementary Fig. S2). It remains unclear whether these differences in gene involvement contribute to variation in IFN signaling activation. Furthermore, whether the observed difference in the response of RECs to live *Mtb* versus HK-*Mtb* exposure is clinically relevant—potentially underlying the recently described manifestations of healed subvascular lesions, focal vascular tortuosity, and occlusive vasculitis, features indicative of a subset of TRV associated with active TB disease^30^—requires further investigation. Although HK-*Mtb* has recently been reported to induce some degree of intraocular inflammation in animal experiments,^31^ we argue that using live, replicating *Mtb* is more relevant for understanding host–pathogen interactions in the study of OTB. An in vitro infection model with live *Mtb* might better recapitulate the mechanisms of TRV as an active disease and a form of extrapulmonary TB.

The findings of our study, which highlight the different cellular responses between live *Mtb*-infected and HK-*Mtb*–exposed RECs, could provide valuable insights in the current understanding of TRV. Currently, pathogen identification in the diagnosis of ocular OTB, such as PCR testing from ocular fluid specimens, is seldom performed, and its results, whether positive or negative, seem to have little influence on clinical management.^32^ Treatment decisions primarily rely on suggestive clinical findings and systemic corroborative investigations, including chest radiology and TB immunoreactivity testing.^33^ Unlike with choroidal granuloma manifestation, where uveitis experts strongly recommend the immediate initiation of ATT, the necessity and duration of ATT for TRV remain uncertain.^33^ Additionally, it remains uncertain whether ATT alone^34^ or immunosuppressants alone, without a full course of ATT,^35^ is sufficient to control inflammation in TRV cases. Moreover, a few OTB patients with positive *Mtb* PCR results from ocular fluid specimens, but without signs of active systemic TB, have been reported to respond well to corticosteroids alone, without requiring ATT.^32^ This raises the possibility, aside from false positives due to laboratory or specimen handling issues, that ocular inflammation could potentially be triggered by a response to *Mtb* antigen remnants in the ocular environment, rather than by active infection from viable *Mtb*.^4^ CXCL10/IP-10, along with CXCL9, has been highlighted as having both diagnostic and prognostic value in TB disease.^36^^,^^37^ Therefore, the role of IFN signaling and associated molecules, such as CXCL10/IP-10 and CXCL9, in the diagnosis and treatment monitoring of OTB patients requires further investigation.

We previously reported that the response of RPE cells, to some extent, resembles that of M2 macrophages upon *Mtb* infection, although M2 macrophages exhibited lesser activation of the IFN signaling pathway but higher and more diverse expression levels of other pro-inflammatory cytokines and chemokines.^7^ Here, we have shown a similar pattern of IFN signaling pathway activation in both human RECs and RPE cells upon *Mtb* infection, supporting the idea that these cells may play a role in orchestrating a subsequent immunological response to actively replicating *Mtb*. Interestingly, the cellular responses of these eye-derived cells mirror those of lung alveolar macrophages, a key cell participating in pulmonary TB disease,^38^ even though the latter cells show a weaker IFN signaling pathway activation. In contrast, lung endothelial cells exhibited a somewhat distinct response to *Mtb* infection, indicating that endothelial cells in different organs may exhibit specialized immunological responses. Recent studies have highlighted the role of endothelial cells in immunity, particularly their ability to sense pathogens and trigger subsequent immune responses.^39^ However, endothelial cells are heterogeneous across various organs, and these organ-specific differences likely contribute to the variations in their immunological response capabilities.^40^ In clinical practice, retinal vasculitis is known to be associated with systemic diseases, such as autoimmune or autoinflammatory disorders, but it rarely coexists with systemic vasculitis.^41^ In the latter, different mechanisms may contribute to vasculitis, as it may involve distinct pathophysiological processes, including immune complex formation, specific anti-neutrophil cytoplasmic antibodies, and impaired intracellular defense mechanisms,^42^ which differ from what we studied. Nevertheless, a significant number of patients with TRV showed no evidence of TB lesions or vasculitis at other sites, yet they still responded effectively to ATT.^15^ Taken together, these observations suggest that human RECs are highly capable of initiating immune response upon infection, which may help to explain the occurrence of isolated retinal vasculitis in TRV patients.

Our study has several limitations. First, due to the technical constraints of our current BSL-3 laboratory, we were unable to acquire three-dimensional images, hampering us from precisely localizing the internalized *Mtb* within the cells during our experiments. Based on our previous studies, we found that two washes without the addition of specific antibiotics after a 24-hour incubation period were sufficient to remove non-internalized *Mtb*.^7^ Second, the RNA quality was suboptimal for standard RNA sequencing methods. However, the QuantSeq 3′ mRNA-Seq approach employed in this study proved adequate for generating transcriptomic profiles aligned with our research objectives.^43^ Furthermore, the elevated levels of CXCL10/IP-10 in the culture supernatants of RECs infected with live *Mtb* further corroborate activation of the IFN signaling pathway, as identified through our transcriptomic data analysis. Third, our analysis is solely based on a single time point (24 hours post-infection). The elevation of several proteins in HK-*Mtb*–exposed RECs, despite the absence of corresponding DEGs in the RNA-seq data, may suggest that the genes associated with these proteins were transiently upregulated prior to the 24-hour time point. Also, we were unable to determine whether the observed type 1 IFN activation impacts TB disease progression, nor could we confirm if modulating this IFN signaling pathway would alter the intracellular fate of *Mtb* in human RECs. A previous study utilizing THP-1 cells demonstrated that intracellular replication of *Mtb* was markedly reduced following treatment with an anti–IFN-α neutralizing antibody, but not with an anti–IFN-β neutralizing antibody. This reduction could be reversed with anti–IFN-γ neutralizing antibodies.^24^ Finally, our pathway comparison between cells derived from eye (RPE cells and RECs) and lung upon *Mtb* infection requires further validation studies. Such in vitro experiments are ideally performed in parallel for optimal comparison.

In conclusion, this study showed that *Mtb* can infect human REC in vitro, which triggers a robust activation of IFN signaling, closely resembling the response in *Mtb*-infected RPE cells. In contrast, exposure to HK-*Mtb* led to the expression of several pro-inflammatory cytokines and chemokines, without further signs of IFN signaling. The cellular response of human RECs to live *Mtb* infection may play a role in the recruitment and activation of leucocytes and contribute to TRV in OTB.

## Supplementary Material

Supplement 1

Supplement 2

Supplement 3
