# Supplementary material for: Mycobacterium tuberculosis Infection of Retinal Endothelial Cells Induces Interferon Signaling Activation: Insights Into Tubercular Retinal Vasculitis
Source: Invest Ophthalmol Vis Sci. 2025 Jul 16;66(9):48. doi: 10.1167/iovs.66.9.48 (PMC12279072; doi:10.1167/iovs.66.9.48)
Supplement: Supplement 1 [file iovs-66-9-48_s001.pdf]

Supplementary Materials

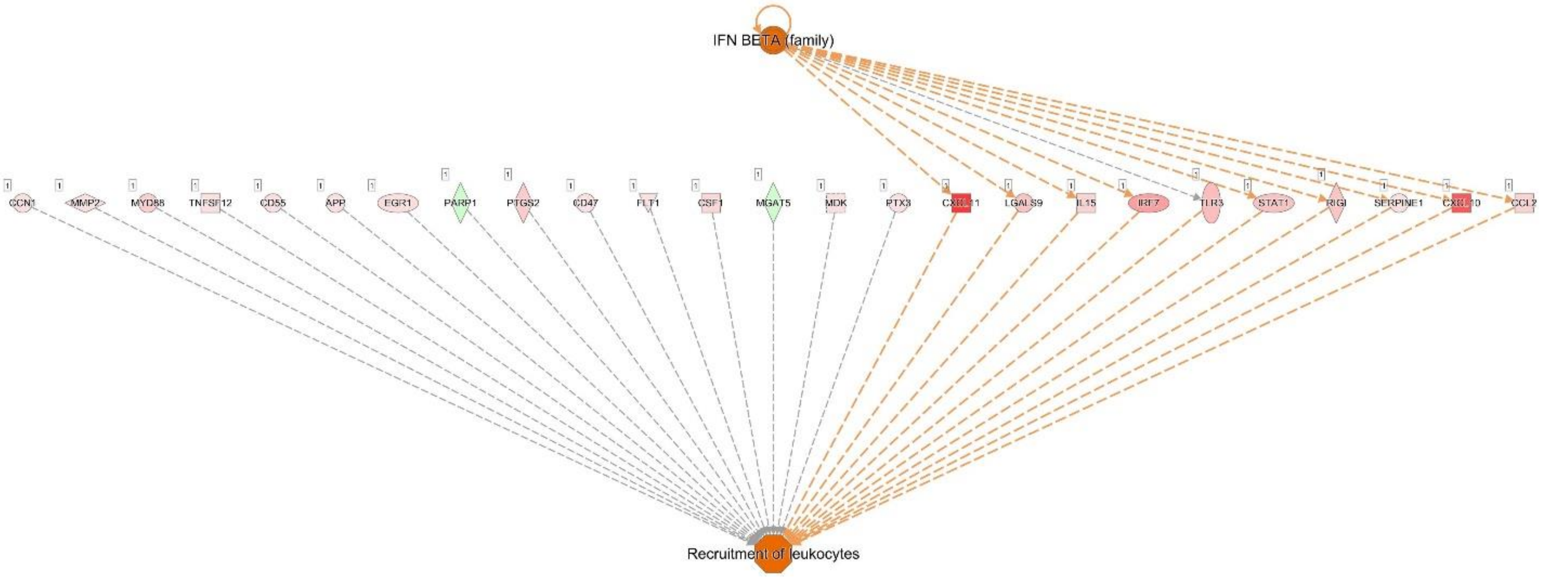

Supplementary Figure 1. Identification of interferon-β as one of predicted regulator effects in human RECs following live *Mtb* infection. Symbols depict the molecules and the coloring indicates the following: Red molecules measured at increased level, green molecules measured at decreased level, and orange molecules predicted to be activated. Orange line indicates activating effect and gray line indicates an unpredictable effect. Solid lines indicate direct effect and dashed lines indicate indirect effect.
The network was generated through the use of QIAGEN IPA (QIAGEN Inc., https://digitalinsights.qiagen.com/IPA).

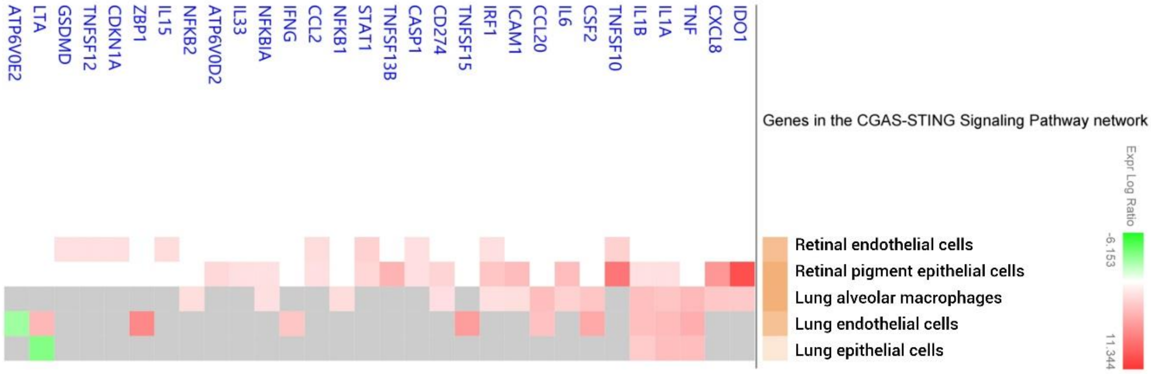

Supplementary Figure 2. Gene heat maps in the cGAS-STING signaling pathways activation from *Mtb* experiments using retinal endothelial cells (current study), retinal pigment epithelial cells (La Distia Nora et al), and lung-derived cells (lung endothelial cells, lung epithelial cells, and lung alveolar macrophages derived from Maertzdorf *et al.* (GSE112483)).
The heat map was generated through the use of QIAGEN IPA (QIAGEN Inc., https://digitalinsights.qiagen.com/IPA).
