# Supplementary material for: Mycobacterium tuberculosis Infection of Retinal Endothelial Cells Induces Interferon Signaling Activation: Insights Into Tubercular Retinal Vasculitis
Source: Invest Ophthalmol Vis Sci. 2025 Jul 16;66(9):48. doi: 10.1167/iovs.66.9.48 (PMC12279072; doi:10.1167/iovs.66.9.48)
Supplement: Supplement 2 [file iovs-66-9-48_s002.pdf]

Supplementary Table 1. Primer-probes set used in RT-PCR

**Gene**	
ABL1	TaqMan Gene Expression Assays (Hs99999002_mH), Life technologies
TLR8	TaqMan Gene Expression Assays (Hs00152972_m1), Life technologies
FCGR1B	TaqMan Gene Expression Assays (Hs00417598_m1), Life technologies
GBP1	TaqMan Gene Expression Assays (Hs00977005_m1), Life technologies
IFIT2	TaqMan Gene Expression Assays (Hs00533665_m1), Life technologies
IRF7	TaqMan Gene Expression Assays (Hs00185375_m1), Life technologies
My88	TaqMan Gene Expression Assays (Hs01573837_g1), Life technologies
SERPING1	TaqMan Gene Expression Assays (Hs00163781_m1), Life technologies
STAT1	TaqMan Gene Expression Assays (Hs01013996_m1), Life technologies
UBE2L6	TaqMan Gene Expression Assays (Hs01125548_m1), Life technologies
MX1	TaqMan Gene Expression Assays (Hs00895598_m1), Life technologies

ABL = abelson murine leukemia viral oncogene homolog 1, *TLR8* = toll like receptor 8, *FCGR1B* = Fc fragment of IgG receptor Ib, *GBP1* = guanylate binding protein 1, *IFIT2* = interferon induced protein with tetratricopeptide repeats 2, *IRF7* = interferon regulatory factor 7, *My88* = myeloid differentiation primary response 88, *SERPING1* = serpin family G member 1, *STAT1* = signal transducer and activator of transcription 1, *UBE2L6* = ubiquitin/ISG15-conjugating enzyme E2 L6, *MX1* = MX dynamin like GTPase 1

Supplementary Table 2. List of master regulators predicted in IPA® in human RECs following live *Mtb* infection filtered to Z-score < or > 8.5

No	Master Regulator	Molecule Type	Participating regulators	Predicted Activation	Activation z-score	p-value of overlap
1	poly rI:rC-RNA	biologic drug	poly rI:rC-RNA	Activated	10.73	5.93E-114
2	lipopolysaccharide	chemical drug	lipopolysaccharide	Activated	9.949	1.01E-56
3	CGAS	enzyme	CGAS,IFN TYPE 1 (family),IRF3,RELA,STAT1,STING1,TBK1	Activated	9.851	1.1E-73
4	IFNG	cytokine	IFNG	Activated	9.57	7.54E-76
5	Tlr13	other	IRF1,IRF5,IRF7,MAPK (family),NFKB (complex),Tlr13	Activated	9.505	6.95E-79
6	Tlr12	other	IRF1,IRF5,IRF7,MAPK (family),NFKB (complex),Tlr12	Activated	9.505	6.08E-79
7	double-stranded DNA	chemical - endogenous mammalian	APEX1,ATM,CGAS,double-stranded DNA,EIF2AK2,EIF2S1,IFIH1,IKBKE,IRF3,NFKB (complex),RIGI,SQSTM1,STING1,TBK1,TLR (family),TLR3,TLR9,TNF,ZBP1	Activated	9.503	3.93E-58
8	Tlr11	transmembrane receptor	IRF1,IRF5,IRF7,MAPK (family),MYD88,NFKB (complex),Tlr11	Activated	9.466	6.03E-75
9	2'3'-cyclic guanosine monophosphate-adenosine monophosphate	chemical - endogenous mammalian	2'3'-cyclic guanosine monophosphate-adenosine monophosphate,IRF3,NOS3,RELA,STAT1,STING1,TBK1	Activated	9.336	8.1E-67
10	IFN TYPE I RECEPTOR (complex)	complex	IFN TYPE I RECEPTOR (complex),IFNAR1,IFNAR2,IRF1,JAK1,STAT1,TYK2	Activated	9.271	1.06E-96
11	PARP9	enzyme	IRF3,IRF7,P85 PIK3R (family),PARP14,PARP9,PI3K (complex),STAT (family),STAT1	Activated	9.146	6.82E-82
12	IL13-downregulated genes for extracellular proteins	group	IL12A,IL12B,IL13-downregulated genes for extracellular proteins,IL1A,IL1B,TNF	Activated	9.087	3.57E-52
13	adavosertib	chemical drug	adavosertib,BRCA2,CDK1,CDK2,CHAF1A,ERK (family),IRF3,NPM1,STAT1,STING1,TBK1,TRIM28	Activated	9.017	2.2E-78
14	SELENOK	other	IRF3,SELENOK,STAT1,STING1,TBK1	Activated	8.91	6.66E-78
15	TLR5	transmembrane receptor	AKT (family),Casp1,IRF1,IRF5,IRF7,MAPK (family),MYD88,NFKB (complex),P38 MAPK (family),PI3K (complex),PRKCE,RAP1A,TLR5,TRAF6	Activated	8.783	1.6E-70
16	SIX4	transcription regulator	SIX4,STAT1,STING1,TBK1	Activated	8.778	1.91E-69
17	IFNA2	cytokine	IFNA2	Activated	8.775	8.04E-94
18	IL4,IL13-downregulated genes for extracellular proteins	group	CCL2,CXCL8,IL1A,IL1B,IL4,IL13-downregulated genes for extracellular proteins,IL6,TNF	Activated	8.731	1.6E-49
19	IFNA8	cytokine	IFNA8,NFKB (complex),STAT1,STAT4	Activated	8.729	3.17E-51
20	poly dA-dT	chemical reagent	Casp1,IRF3,JNK (family),P38 MAPK (family),poly dA-dT,RELA,STAT1,TBK1,ZBP1	Activated	8.701	8.77E-56
21	lipid A	chemical toxicant	Casp1,ERK1/2 (family),IRF3,JINK1/2 (family),JNK (family),lipid A,STAT1,TLR (family),TLR4	Activated	8.701	2.37E-57
22	TNF	cytokine	TNF	Activated	8.693	9.95E-48
23	aphidicolin	chemical toxicant	aphidicolin,ATM,BRCA1,CDK1,CHEK1,CHEK2,IKBKG,IKK (complex),NFKB (complex),RPS6KB1,SIRT (family),STAT1,TERT	Activated	8.684	9.05E-52
24	LBP	transporter	CD14,ERK1/2 (family),IKB (family),IRF3,JNK (family),LBP,MAPK (family),NFKB (complex),P38 MAPK (family),STAT1,TYK2	Activated	8.644	1.87E-48
25	interferon beta-1a	biologic drug	IFNAR1,IFNAR2,interferon beta-1a,STAT1,STAT2	Activated	8.607	1.27E-92
26	PRKRA	other	EIF2AK2,ESTROGEN RECEPTOR (family),IRF3,IRF7,NR3C1,PRKRA,RAC1,RIGI,STAT1	Activated	8.525	6.75E-73
27	Lilrb2	transmembrane receptor	Lilrb2,NFKB (complex),STAT1,TLR4	Inhibited	-8.556	6.23E-49
28	LAMP2	other	LAMP2,STAT1,STING1,TBK1	Inhibited	-8.778	1.2E-67
29	DNAJA2	enzyme	DNAJA2,STAT1,STING1,TBK1	Inhibited	-8.835	8.53E-71
30	CACTIN	other	CACTIN,IRF3,IRF7,NFKB (complex)	Inhibited	-9.12	4.98E-70
31	DHX58	enzyme	DHX58,IFIH1,IRF (family),IRF3,MAVS,NFKB (complex),PLAAT4,RIGI,STAT1	Inhibited	-9.336	7.93E-65
